# Ultralow Loading of Ru as a Bifunctional Catalyst for the Oxygen Electrode of Solid Oxide Cells

**DOI:** 10.1021/acscatal.3c02544

**Published:** 2023-08-08

**Authors:** Haoyu Li, Hyong June Kim, ThomasJae Garcia, Geonwoo Park, Yong Ding, Meilin Liu, Jihwan An, Min Hwan Lee

**Affiliations:** † Department of Mechanical Engineering, University of California, Merced, California 95343, United States; ‡ Department of Manufacturing System and Design Engineering, Seoul National University of Science and Technology, Seoul 01811, Republic of Korea; § School of Materials Science and Engineering, Georgia Institute of Technology, Atlanta, Georgia 30332-0245, United States; ∥ Department of Mechanical Engineering, Pohang University of Science and Technology (POSTECH), Pohang 37673, Republic of Korea

**Keywords:** solid oxide fuel cell, solid oxide electrolysis cell, plasma-enhanced atomic layer deposition, ruthenium, Sr segregation

## Abstract

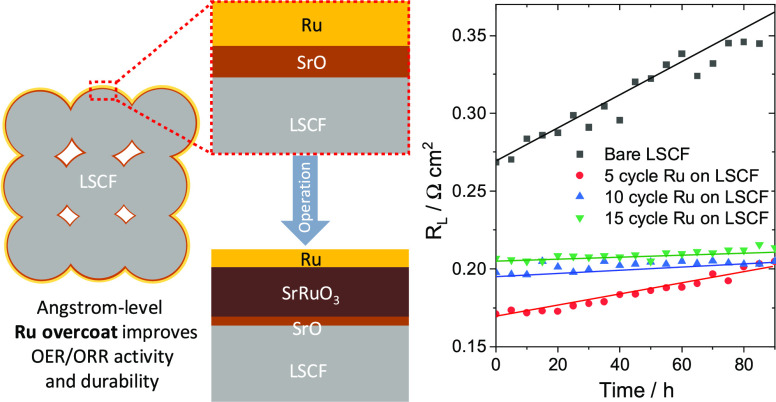

The oxygen evolution reaction (OER) is a significant contributor to the cell overpotential in solid oxide electrolyzer cells (SOECs). Although noble metals such as Ru and Ir have been utilized as OER catalysts, their widespread application in SOECs is hindered by their high cost and limited availability. In this study, we present a highly effective approach to enhance air electrode performance and durability by depositing an ultrathin layer of metallic Ru, as thin as ∼7.5 Å, onto (La_0.6_Sr_0.4_)_0.95_Co_0.2_Fe_0.8_O_3-δ_ (LSCF) using plasma-enhanced atomic layer deposition (PEALD). Our study suggests that the emergence of a perovskite, SrRuO_3_, resulting from the reaction between PEALD-based Ru and surface-segregated Sr species, plays a crucial role in suppressing Sr segregation and maintaining favorable oxygen desorption kinetics, which ultimately improves the OER durability. Further, the PEALD Ru coating on LSCF also reduces the resistance to the oxygen reduction reaction (ORR), highlighting the bifunctional electrocatalytic activities for reversible fuel cells. When the LSCF electrode of a test cell is decorated with ∼7.5 Å of the Ru overcoat, a current density of 656 mA cm^–2^ at 1.3 V in electrolysis mode and a peak power density of 803 mW cm^–2^ in fuel cell mode are demonstrated at 700 °C, corresponding to an enhancement of 49.1 and 31.9%, respectively, compared to the pristine cell.

## Introduction

Solid oxide cells (SOCs) are an electrochemical device that has garnered significant attention due to their high efficiency, fuel flexibility, and reversibility for operation in both fuel cell and the electrolysis modes.^1,2^ However, the relatively high operating temperature (typically > 800 °C) required presents various challenges, including fast degradation, high operating costs, and slow start-up.^3^ To overcome these issues, significant efforts have been made to decrease the operating temperature,^4,5^ but this causes a dramatic decrease in performance due to the low catalytic activity of typical electrode materials. To make the cell performance viable for a wide commercial deployment, a significant enhancement in the electrode performance is still required. Considerable efforts have been dedicated to addressing the challenge of low catalytic activity in oxygen electrodes. One effective strategy involves the application of a surface coating or the introduction of nanoparticles containing highly catalytically active species onto a traditional oxygen electrode backbone.^6−8^ This approach offers flexibility in material choices and inherent simplicity, making it a promising avenue for enhancing electrode performance.

In the context of solid oxide electrolysis cells (SOECs), the oxygen evolution reaction (OER) is the major contributor to the cell overpotential. While Ru- and Ir-based materials are considered the most effective catalysts for OER, their high cost and limited availability make it impractical for widespread deployment of SOECs. To address this, there have been reports, albeit scarce, on the utilization of a small amount of Ru/RuO_2_ as a surface-coating material for air electrodes. Li et al. found that 0.5 wt % RuO_2_ nanodots infiltrated on La_0.5_Ba_0.25_Sr_0.25_Co_0.8_Fe_0.2_O_3-δ_ (LBSCF)-based air electrode significantly improved the OER kinetics, particularly for low-frequency processes such as molecular transport and dissociation during OER.^9^ Similarly, Song et al. formed 6 wt % RuO_2_ nanoparticles on strontium-doped lanthanum manganite/yttria-stabilized zirconia (LSM/YSZ) composite through infiltration, resulting in an enhancement of cell current density from 0.46 to 0.74 A cm^–2^ at 1.2 V and 800 °C.^10^

In this report, we first demonstrate a significant enhancement of air electrode performance by depositing an ultralow loading of metallic Ru onto the (La_0.6_Sr_0.4_)_0.95_Co_0.2_Fe_0.8_O_3-δ_ (LSCF) air electrode using plasma-enhanced atomic layer deposition (PEALD). Atomic layer deposition (ALD) is an emerging chemical deposition technique capable of creating highly uniform nanodots or thin films, even on complex geometries, with precise control over thickness and composition by leveraging its self-limiting deposition characteristics.^11,12^ ALD has already shown success in solid oxide fuel cell (SOFC) applications, improving cell performance and durability through the creation of uniform atomic-scale overcoats.^7,13^ Our earlier work demonstrated that angstrom-level metal oxides enhance oxygen reduction reaction (ORR) activity, prevent thermal agglomeration, and adjust cation segregation in SOFCs.^14−17^ The PEALD, an ALD variant we employed for this study, utilizes plasma to enhance the reaction kinetics between precursors with the substrate surface, resulting in improved film quality and a broader range of deposition options.^18^

In addition to the beneficial effect of PEALD Ru on OER activity, our findings demonstrate that the perovskite (SrRuO_3_), formed through the reaction between ALD-based Ru and surface-segregated Sr species, plays a role in mitigating Sr segregation and favorable oxygen desorption kinetics, contributing to the long-term durability of OER. Lastly, we also discuss the effect of ALD Ru on the enhancement of ORR performance, suggesting its potential as a bifunctional air electrode for reversible fuel cells. To our knowledge, this is the first report on the use of ALD for metallic Ru deposition on air electrodes in either fuel cell or electrolysis mode.

## Experimental Section

### Cell Preparations

Each cell was diced from a commercialized anode-supported half-cell (Kceracell, 11 cm × 11 cm) to a dimension of 2 cm × 2 cm. The cell consists of a 600 μm NiO-YSZ composite support, a 30 μm NiO-YSZ functional layer, and a 5 μm dense YSZ electrolyte. For the air electrode, (La_0.6_Sr_0.4_)_0.95_Co_0.2_Fe_0.8_O_3-σ_ (LSCF) is used, and a GDC interlayer (20 mol % Gd) is placed between the YSZ and LSCF to prevent the formation of zirconates^19,20^ and improve contact between the electrode and the electrolyte.^21^ To deposit the GDC interlayer, a GDC slurry is first prepared by mixing Hypermer KD-1 (Croda, dispersant) for 24 h at 50 °C. GDC nanopowders (FuelCellMaterials; 20 mol % GDC; surface area: 35.3 m^2^ g^–1^) and ethyl cellulose (Sigma-Aldrich, binder) are then added to the mixture and stirred for another 24 h at 50 °C. The final slurry is composed of 40 wt % terpineol, 10 wt % Hypermer KD-1, 2 wt % ethyl cellulose, and 48 wt % GDC nanopowder. LSCF slurry is prepared using the same method as the GDC slurry but with different compositions. It consists of 35 wt % of terpineol, 5 wt % of Hypermer KD-1, 3 wt % of ethyl cellulose, and 57 wt % of LSCF powers (FuelCellMaterials). After preparing the slurry, the GDC slurry is screen-printed on the YSZ side and then sintered at 1150 °C for 5 h at a rate of 3 °C min^–1^, with additional stops at 80 °C for 1 h and 500 °C for 30 min to evaporate the solvent and binders, respectively. LSCF slurry is then screen-printed on the GDC and sintered at 850 °C for 3 h, with the same thermal procedure as that of GDC deposition.

On the top of the LSCF electrode, an atomic-scale metallic Ru is coated by PEALD in a custom-built chamber. (Carbonyl cyclohexadiene)Ru is utilized as the Ru precursor, while hydrogen plasma (150 W RF sputtered source, 400 mTorr plasma pressure) is used as the reactant. Argon (Ar) is used as the carrier gas and purging gas with a constant flow rate of 50 sccm. The canister temperature for Ru is 45 °C, and the chamber temperature is 250 °C. One ALD cycle is comprised of Ru precursor pulse (1 s), precursor diffusion (10 s), purging (30 s), H_2_ plasma exposure (10 s), and purging (30 s). The growth rate for the metallic Ru is ∼1.5 Å cycle^–1^; details of the Ru PEALD process are provided in previous work.^22^

A separate set of the sample was prepared for X-ray diffraction (XRD) analysis to reveal the chemical properties of the LSCF backbone without being obscured by the presence of YSZ, GDC, and NiO. To make LSCF pallets for this purpose, LSCF powders was first ball-milled and pressed under a uniaxial press, followed by a sintering process at 850 °C for 10 h. To investigate the crystallinity of PEALD Ru by XRD, a sample with 300 cycles of Ru PEALD on a Si wafer was used. Transmission electron microscopy (TEM) samples were prepared by grinding LSCF with a Ru overcoat into powders, dissolving into ethanol, and drop-casting the particle suspension upon a 3 mm lacey-carbon grid (TED Pella).

### Physical Characterization

A field-emission scanning electron microscope (FE-SEM, Zeiss Gemini 500) was used at 3 kV to observe the microstructure. X-ray photoelectron spectroscopy (XPS) was performed on a Nexus system (Thermo Fisher Scientific) using monochromated, micro-focused, low-power Al Kα X-ray source for excitation and a 180°, double-focusing, hemispherical analyzer with a 128-channel detector (10–400 μm spot size with adjustable sample holder incident to the X-ray beam from 0 to 60°). The phase and composition of LSCF were evaluated by XRD using a PANalytical X’Pert Pro system with Co-Kα radiation (λ = 1.7890 Å). The crystallinity of PEALD Ru was evaluated by GIXRD (Smart Lab, Rigaku Corporation) with Cu Kα radiation (λ = 1.5406 Å). All of the XRD data are converted to Cu Kα radiation-based angles. A Hitachi HD2700 aberration-corrected scanning transmission electron microscope was used to record the high-angle annular dark-field (HAADF) STEM images.

### Electrochemical Characterization

Electrochemical characterization was performed by electrochemical impedance spectroscopy (EIS; Bio-Logic SP-200) with 20 mV of AC perturbation. Cell performance was measured with a scan rate of 20 mV s^–1^ for both fuel cell and electrolysis modes. A Pt mesh (GoodFellow) was used as the current collector for the air electrode, while porous Ni foam served as the current collector for the fuel electrode. A 3 kg load was applied through the cell to ensure solid contact between the electrodes and the current collecting mesh/foam. The distribution of relaxation time (DRT) calculation relies on Tikhonov regularization, which involves the discretization of continuous functions.^23^

The cell is first heated to 700 °C at a rate of 2.5 °C min^–1^, while dry H_2_ is supplied at the fuel electrode with a flow rate of 100 sccm. The cell is continuously reduced until the open circuit voltage (OCV) of the cell is stabilized at ∼1 V. After NiO on the fuel side was completely reduced to Ni metal, fuel cell performance was initially investigated at different operating temperatures with 100 sccm O_2_ on the air electrode and 100 sccm H_2_ on the fuel electrode.

After the electrochemical testing in fuel cell mode, the cells were then tested in electrolysis mode at the same temperature, 700 °C. On the fuel electrode, H_2_ was fed through a water bath as a reduced flow rate of 50 sccm. Relative humidity was controlled at ∼50% by adjusting the temperature of the water bath to 82 °C. The entire fuel channel was covered by a heating tape (160 °C at the inlet and 170 °C at the outlet) to prevent the condensation of steam. On the air electrode, O_2_/N_2_ with a ratio of 21:79 was supplied to simulate the constant flow of ambient air with a total flow rate of 100 sccm. Durability tests in electrolysis mode were performed at a constant current density of 500 mA cm^–2^. I–V and EIS measurements were performed every 5 h.

## Results and Discussion

**Figure 1:**
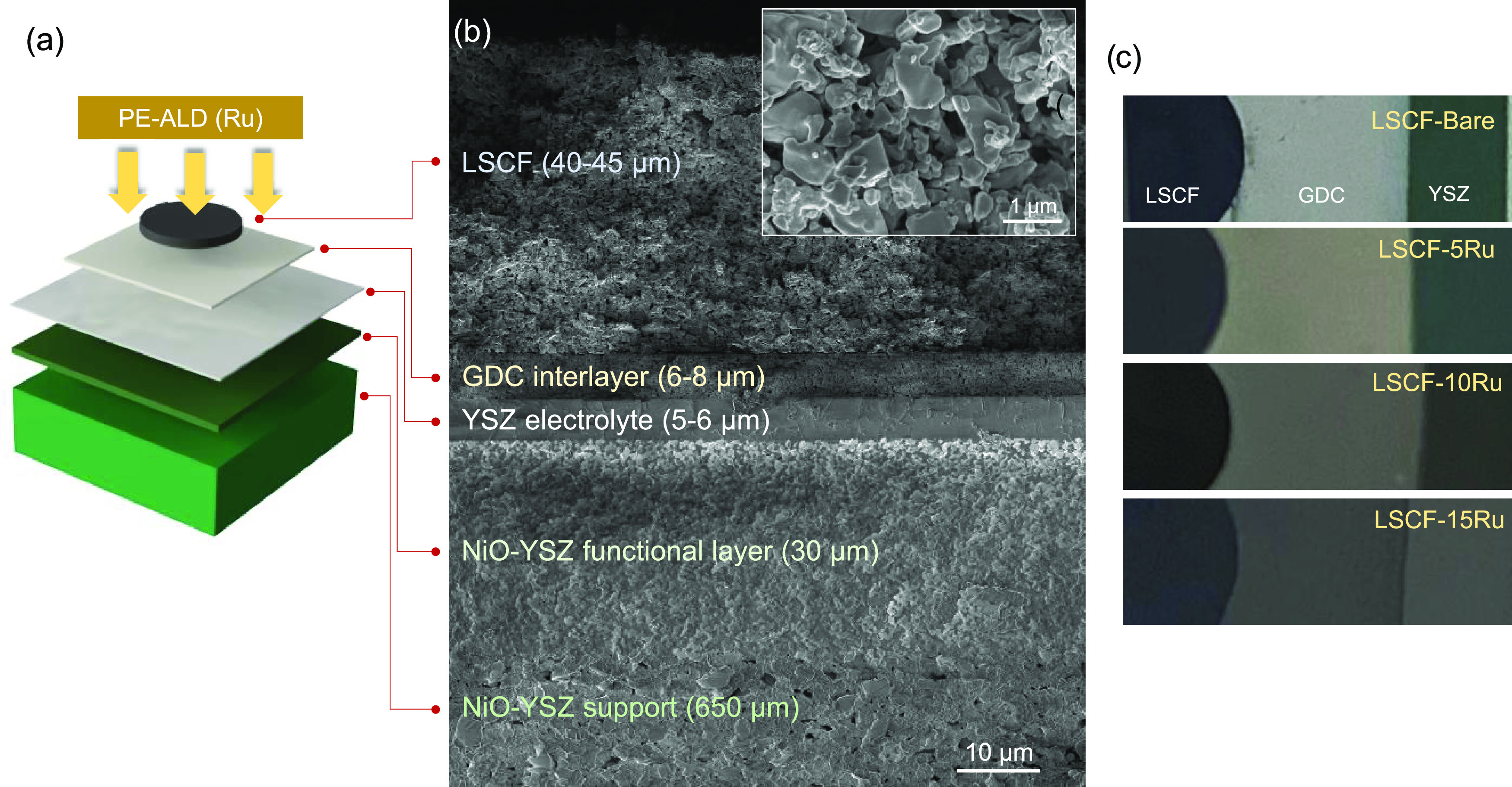
(a) Schematic diagram of the cell configuration. (b) Cross-sectional SEM image of a LSCF-Bare cell. The inset shows a zoomed-in image of the LSCF layer. (c) Top-view optical images of as-prepared cells.

A full cell in the configuration of LSCF/GDC/YSZ/NiO-YSZ is prepared as detailed in the Experimental Section; a cross-sectional SEM micrograph is presented in Figure 1b. To examine the impact of the Ru overcoat, an ultrathin layer of metallic Ru species is deposited onto the surface of the LSCF backbone using PEALD, as depicted in Figure 1a. Four thickness levels are prepared by performing 0, 5, 10, and 15 cycles of Ru PEALD, resulting in cells named LSCF-Bare, LSCF-5Ru, LSCF-10Ru, and LSCF-15Ru, respectively. A separate characterization of Ru PEALD on a silicon wafer substrate reveals that Ru is uniformly deposited over the surface at a nominal growth rate of ∼1.5 Å cycle^–1^.^22^ The XRD spectra in Figure S1 reveal that the metallic Ru deposited by PEALD comprises a hexagonal structure (*P*63/*mmc* space group), while the backbone contains mostly rhombohedral LSCF (*R*3*m* space group) with a minor presence of an impurity phase. Considering the majority of ORR and OER occurring at the electrode region in the vicinity of the electrode/electrolyte interface, a rather thin (40 μm) LSCF electrode with high porosity is utilized as the electrode backbone to ensure that sufficient ALD reaction occurs around the interface. Figure 1c shows the top-view optical images of the as-prepared cells, showing an increasingly prominent metallic gleam on the surface with a larger number of cycles for the Ru PEALD overcoat. The Ru content in the LSCF-5Ru sample is quantified and estimated to be ∼1.33 wt % at its maximum value; see Estimation of Ru wt % of LSCF-5Ru in the Supporting Information.

**Figure 2:**
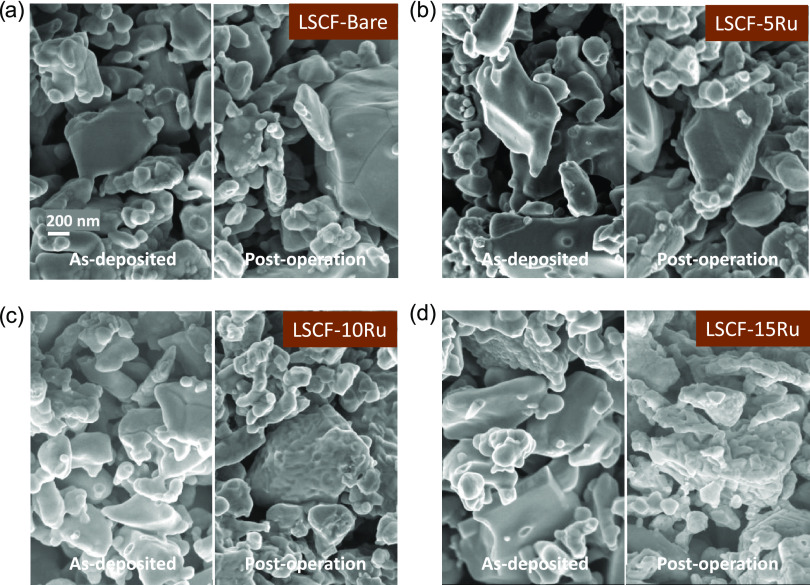
SEM image of LSCF-Bare (a), LSCF-5Ru (b), LSCF-10Ru (c), and LSCF-15Ru (d) cells before and after a 90 h durability test under electrolysis mode (500 mA cm^–2^ at 700 °C).

SEM images were utilized to investigate the surface structure of cells both before and after extended use in the electrolysis mode, as presented in Figure 2. Results indicate that the Ru PEALD overcoat consistently maintained a smooth and uniform appearance over the LSCF electrode in all as-deposited cells, without any discernible features caused by the ALD process. However, after a 90 h long electrolysis test at 700 °C under 500 mA cm^–2^, the cell surface displayed additional features, which were more pronounced with a thicker Ru overcoat. Particularly in the case of LSCF-15Ru, the additional layer exhibits nanoparticle-like features ranging in size from 100 to 200 nm. It is noted that such features were not observed in cells without a Ru overcoat, suggesting that the changes in morphology are associated with the presence of the Ru layer.

**Figure 3:**
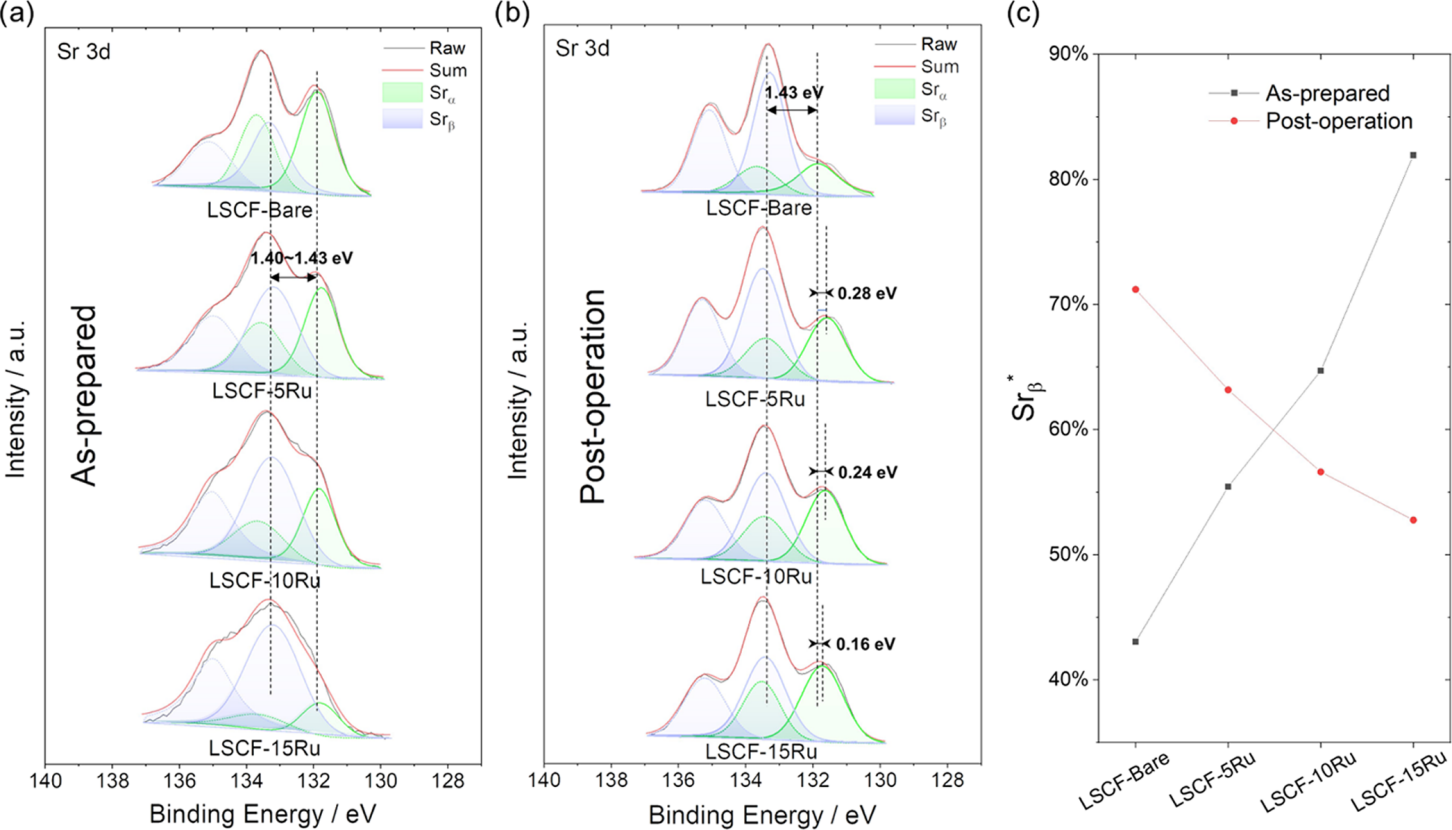
(a, b) Sr 3d XPS spectra of as-prepared samples (a) and post-operation samples (b). Post-operation samples are those that underwent a 90 h long test at 500 mA cm^–2^ in electrolysis mode. (c) Resultant *Sr*_β_^*^ values for each sample.

XPS analysis was performed to examine the surface chemistry of the four different air electrodes before and after the 90 h long operations in electrolysis mode. First, Sr 3d spectra are deconvoluted into two distinct peaks: Sr_α_ (131.8–132.1 eV) and Sr_β_ (133.3–133.6 eV). Sr_α_ corresponds to Sr atoms residing within the lattice of the perovskite structure, while Sr_β_ is attributed to Sr on the surface of the structure.^24,25^ This surface Sr is associated with various compounds including SrO, Sr(OH)_2_, and SrCO_3_.^26^ The relative quantities of lattice Sr and surface Sr, which are quantified by [*Sr*_α_]/([*Sr*_α_] + [*Sr*_β_]) and [*Sr*_β_]/([*Sr*_α_] + [*Sr*_β_]), are denoted as *Sr*_α_^*^ and *Sr*_β_^*^, respectively. For the as-prepared samples, as the thickness of the Ru overcoat increases, the Sr_β_ peak becomes dominant (Figure 3c). This observation makes sense when considering that as the Ru overcoat increases in thickness, the depth at which XPS can effectively detect the LSCF lattice becomes shallower. As a result, surface Sr contributes more to the detected signal compared to lattice Sr.

**Figure 4:**
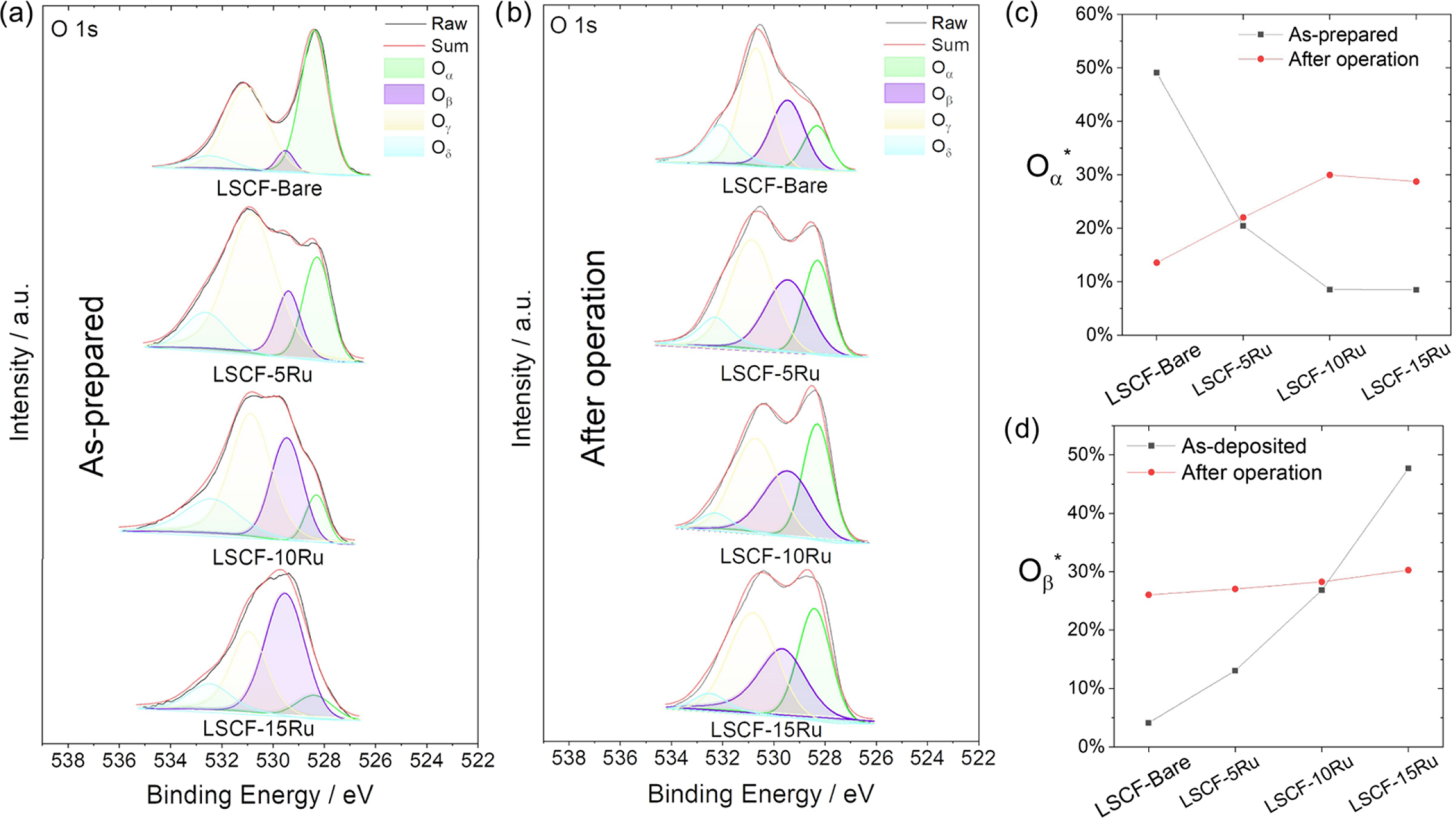
(a, b) O 1s XPS spectra of as-prepared samples (a) and post-operation samples (b). Post-operation samples are those that underwent a 90 h long test at 500 mA cm^–2^ in electrolysis mode. (c, d) Resultant *O*_α_^*^ (c) and *O*_β_^*^ (d) values for each sample.

The O 1s spectra shown in Figure 4 are also aligned well with this interpretation. The peaks at ∼528.3, 530.0, 530.9, and 532.4 eV are ascribed to lattice oxygen (namely, *O*_α_), oxygen defect (*O*_β_), surface-adsorbed oxygen species (*O*_γ_), and surface water (*O*_δ_), respectively.^15,27−30^ The relative quantities of lattice oxygen and oxygen vacancy are quantified by [*O*_α_]/[*O*_sum_] and [*O*_β_]/[*O*_sum_] and named as *O*_α_^*^ and *O*_β_^*^, respectively, where [*O*_sum_] = [*O*_α_] + [*O*_β_] + [*O*_γ_] + [*O*_δ_]. In the case of the as-prepared cells, a thicker Ru overcoat leads to a decrease in *O*_α_^*^ (Figure 4c), which can be explained again by the fact that as the Ru overcoat becomes thicker, the LSCF lattice contributes less to the XPS signal due to the limited depth of XPS detection. This interpretation is reinforced by the observation that the overall amount of La, Sr, Co, and Fe decreases significantly as the Ru overcoat becomes thicker, as shown in Figure S2.

However, after the prolonged electrolysis mode operation, the opposite trend becomes evident; with a thicker Ru overcoat, fewer surface Sr species (as represented by *Sr*_β_^*^) and more lattice O species (as represented by *O*_α_^*^) compared to the bare sample.Furthermore, after the 90 h operation, the Sr_α_ peaks of Ru-overcoated cells shift to lower binding energy levels, suggesting a notable change in the atomic arrangement surrounding Sr species. These observations all point to the possibility of forming a new Sr-containing oxide phase onto the Sr-segregated LSCF surface during the electrolysis mode operation, which is evidenced by the TEM images shown below.

**Figure 5:**
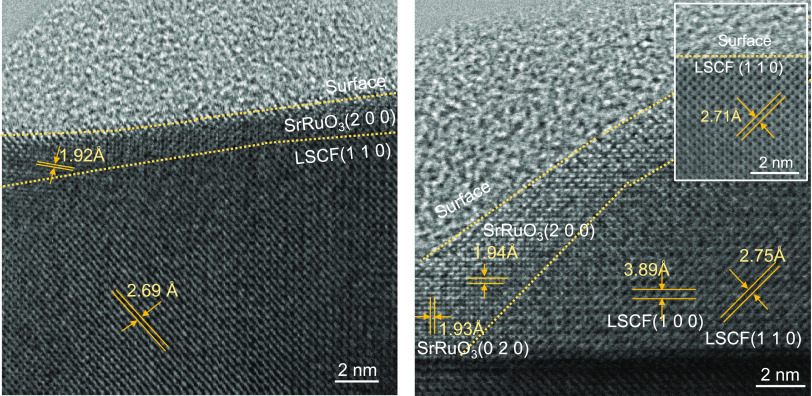
High-resolution TEM images of LSCF-15Ru after the durability test in electrolysis mode for 90 h. The two micrographs are from different locations of the same sample. The one in the inset is from a location without the SrRuO_3_ overcoat in the same sample.

Figure 5 presents high-resolution TEM micrographs of LSCF-15Ru after 90 h long operation in electrolysis mode at 700 °C. The micrographs clearly illustrate the formation of a conformal layer on the surface of the LSCF electrode. The observed spacing in the surface layer, ∼1.93 Å, suggests the presence of the (2 0 0) plane of SrRuO_3_. Although the lattice constant of SrRuO_3_ closely resembles that of LSCF, the atoms in the surface layer appear brighter. This difference in brightness between the surface layer and LSCF can be attributed to the incorporation of heavy Ru atoms within the surface layer. The thickness of the newly formed perovskite overcoat, measuring 2–3 nm, is reasonable, considering that the initial Ru thickness in the as-prepared LSCF-15Ru is expected to be ∼2.25 nm.

**Figure 6:**
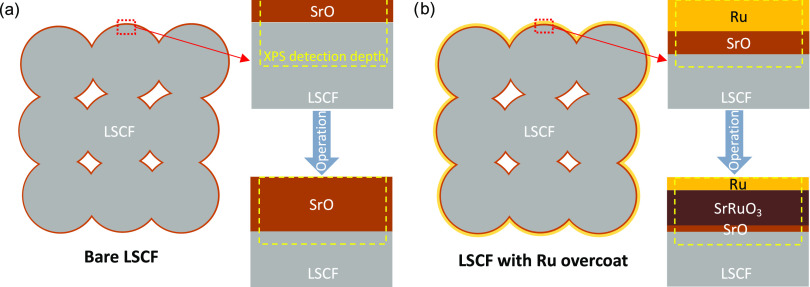
Schematic diagrams depicting cross-sectional views of (a) a LSCF-Bare cell and (b) a Ru-overcoated cell before and after a 90 h long operation in electrolysis mode. Given that the detection depth of XPS is conjectured significantly smaller than the average particle size of LSCF, the morphological effect of the LSCF backbone in XPS analysis is deemed insignificant.

The interpretation of the XPS and TEM characterization can be summarized using the schematic diagrams depicted in Figure 6. The sample without an Ru overcoat (Figure 6a) shows a significant Sr segregation after the durability test, which is supported by the increase in *Sr*_β_^*^ and decrease in *O*_α_^*^. On the other hand, as-prepared cells with a Ru overcoat (Figure 6b) show a higher *Sr*_β_^*^ compared to the case without an overcoat (Figure 6a) because of a decrease in the relative concentration of lattice Sr within the XPS detecting area. However, in the post-operation cells, the surface Sr-including phase (e.g., SrO) is conjectured to have reacted with Ru and formed a new perovskite, SrRuO_3_.

**Figure 7:**
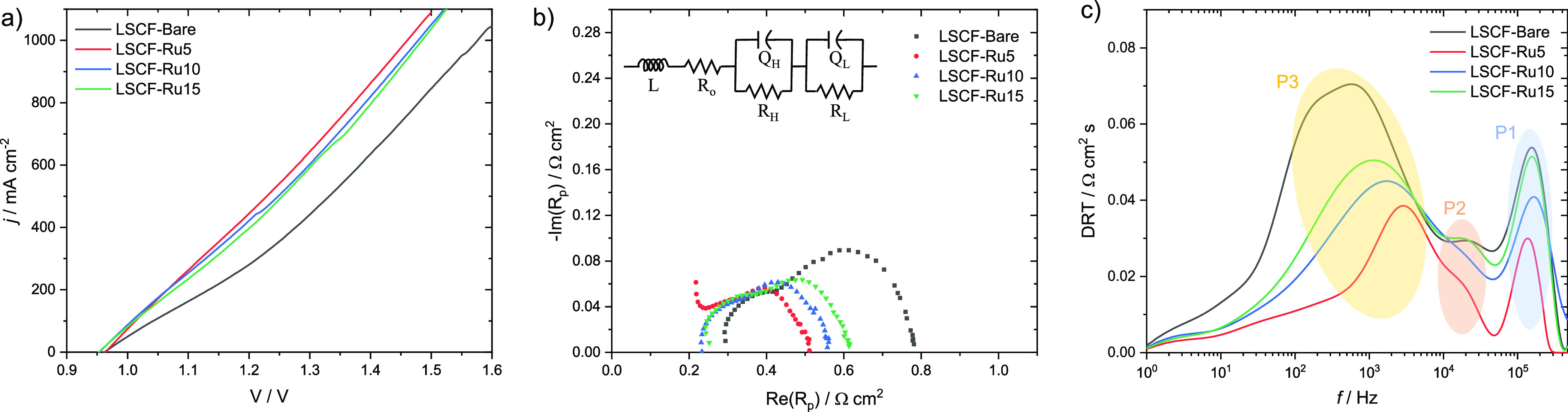
Electrochemical tests of as-prepared cells in electrolysis mode. (a) Polarization curves, (b) EIS curves obtained at 0.97 V along with the equivalent circuit used for fitting, and (c) DRT curves. All of the tests were performed at 700 °C.

Electrochemical data in electrolysis mode was obtained at 700 °C for both as-preparation cells (Figure 7) and post-operation cells (Figure S5). The reversible potential of the samples ranged 0.95–0.97 V, consistent with other SOEC studies using a 50% relative humidity H_2_ flow.^31,32^ LSCF-5Ru exhibited the best performance with a current density of 656 mA cm^–2^ at 1.3 V, significantly outperforming LSCF-Bare (440 mA cm^–2^ at 1.3 V). It is noted that a Ru overcoat with more than 5 ALD cycles resulted in decreased performance. This is conjectured that an excessive ALD overcoat acts as a barrier against oxygen ions, while they may afford a more active OER electrocatalytic kinetics.

To gain further insight into the electrochemical processes occurring in the samples, EIS was performed, and the resulting data were analyzed using an equivalent circuit shown in Figure 7b. The Ohmic resistance (*R*_O_), representing the ionic movement within the electrolyte material and through the interface between the electrolyte and electrodes, was similar for all three Ru-overcoated cells (∼0.20 Ω cm^2^ at 700 °C) but smaller than that of LSCF-Bare (∼0.25 Ω cm^2^). The decrease in *R*_O_ by a Ru overcoat can be attributed to a decrease in interlayer contact resistances (at GDC/LSCF and LSCF/Pt mesh interfaces), in the electronic resistance through the LSCF electrode, or both. The process corresponding to *R*_H_//Q_H_, occurring in the high *f*_c_ range of 10^4^–10^5^ Hz, is attributed to the charge transfer process, whereas the process of *R*_L_//*Q*_L_ occurring in the *f*_c_ range of 10^2^–10^4^ Hz is associated with the oxygen desorption process. LSCF-5Ru shows the lowest electrode resistances in both *R*_H_ (0.08 Ω cm^2^) and *R*_L_ (0.17 Ω cm^2^ at 700 °C), while the values of *R*_H_ and *R*_L_ increased with a thicker Ru overcoat. The P2 and P3 peaks in the DRT plots (Figure 7c) that correspond to *R*_H_//Q_H_ and *R*_L_//*Q*_L_, respectively, were significantly reduced by the Ru overcoat, further indicating the advantageous effect of Ru on both charge transfer and oxygen desorption kinetics. There were no prominent peaks in the *f*_c_ range of <10^2^ Hz, suggesting that mass transport does not pose a bottleneck in electrolysis mode.

**Figure 8:**
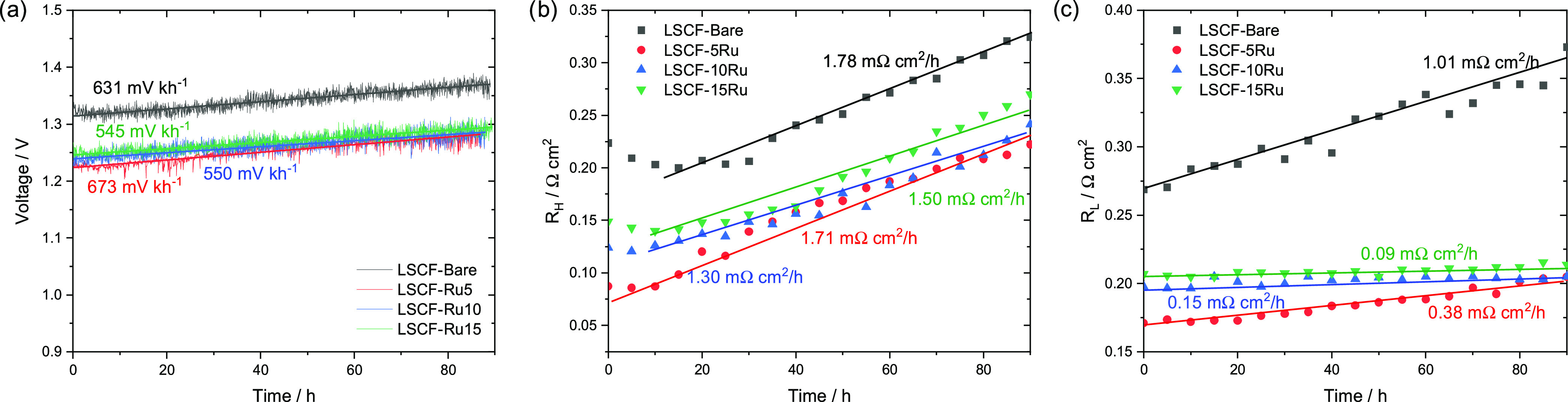
(a) Galvanostatic cell durability tests in electrolysis mode at 500 mA cm^–2^. (b, c) *R*_H_ and *R*_L_ quantified from EIS measurements at different stages of the cell durability test in electrolysis mode at 500 mA cm^–2^. The EIS measurements were performed at 0.97 V every 5 h.

To investigate the evolution of the effectiveness of the Ru overcoat during high-temperature operation in electrolysis mode, a prolonged galvanostatic test was conducted at 500 mA cm^–2^ and 700 °C for 90 h. As shown in Figure 8a, all cells exhibit a nearly linear degradation pattern. LSCF-Bare demonstrated a degradation rate of 631 mV kh^–1^, while cells with thicker Ru overcoats of 5, 10, and 15 cycles exhibited decreasing degradation rates of 673, 550, and 545 mV kh^–1^, respectively. The slightly faster degradation of LSCF-5Ru can be attributed partially to the loss of the active Ru/RuO*_x_* site on the electrode surface, as indicated by XPS analysis (Figure S4). However, cells with thicker Ru overcoats (LSCF-10Ru and LSCF-15Ru) exhibited improved stability compared to the LSCF-Bare cell. This improved stability is attributed to the formation of 2–3 nm thick SrRuO_3_, as observed in the TEM image (Figure 6). It is conjectured that the thin perovskite film has contributed to suppressing Sr segregation toward the LSCF surface, as demonstrated by the XPS results on post-operation samples (Figure 3c).

In addition, EIS measurements were performed every 5 h during the 90 h durability test to better understand the evolution of *R*_H_ and *R*_L_ values at each stage of galvanostatic operation. In terms of *R*_H_, LSCF-10Ru (1.30 Ω cm^2^ kh^–1^) and LSCF-15Ru (1.50 Ω cm^2^ kh^–1^) displayed a somewhat slower degradation rate compared to LSCF-Bare (1.78 Ω cm^2^ kh^–1^), while LSCF-5Ru (1.76 Ω cm^2^ kh^–1^) exhibited a similar degradation rate as LSCF-Bare. The surface area of the Ru-overcoated samples seemed to increase after the durability test, as shown in the SEM-based surface morphology (Figure 2), indicating that the typical trend of surface area decrease due to electrode agglomeration is unlikely to have played a role in the cell performance degradation. Therefore, the degradation in these cells is mainly attributed to the surface segregation of Sr species.^33,34^ Despite the enhancement of electrode performance by the high catalytic activity of Ru/RuO*_x_* toward OER, as evidenced by the significantly lower *R*_H_ values of Ru-coated samples compared to LSCF-Bare, the presence of the active Ru/RuO_x_ phase was observed to decrease, as shown in the XPS results (Figure S4b), likely due to possible evaporation or the formation of a secondary perovskite phase (SrRuO_3_), leading to cell performance degradation. The slightly deaccelerated degradation in LSCF-10Ru and LSCF-15Ru is attributed to the formation of SrRuO_3_, which is likely to have beneficially contributed to the electrode performance by suppressing the surface segregation of Sr. On the other hand, for *R*_L_, the degradation rate of Ru-overcoated cells (0.09–0.38 Ω cm^2^ kh^–1^) decreased with thicker Ru overcoats, and LSCF-15Ru (0.09 Ω cm^2^ kh^–1^) shows a negligible degradation rate compared to LSCF-Bare (1.01 Ω cm^2^ kh^–1^). This suggests that the Ru overcoat is highly effective in maintaining the oxygen desorption kinetics. For all samples, the Ohmic resistances were consistently maintained during the operation (Figure S11), indicating that no substantial secondary phase has developed at the interface of the electrode and the electrolyte.

Here is a proposed mechanism behind the enhanced durability conferred by the Ru overcoat. In the LSCF lattice, Sr species (*Sr*_*La*_^’^) tend to undergo surface segregation, likely driven by electrostatic interactions with enriched surface oxygen vacancies (*V*_*O*_^··^) and/or the elastic energy resulting from the size mismatch between Sr and La species.^33,34^ This segregated Sr can then react with neighboring oxygen or hydroxide species, resulting in the formation of an insulating layer of SrO/Sr(OH)_2_. However, when a SrRuO_3_ blocking layer is introduced atop LSCF, the migration of Sr species to the surface becomes difficult due to the stable Sr species within SrRuO_3_ and the resulting high energy barrier for Sr migration through SrRuO_3_. Furthermore, the closely matched lattice constants of SrRuO_3_ and LSCF (0.394 and 0.393 nm, respectively) leave little room for elastically driven migration. At the interface of SrRuO_3_ and LSCF, there is a scarcity of additional oxygen or hydroxides, preventing the further formation of SrO/Sr(OH)_2_. Consequently, the factors promoting Sr segregation are significantly reduced by the presence of the Sr-rich perovskite layer on the surface.

**Figure 9:**
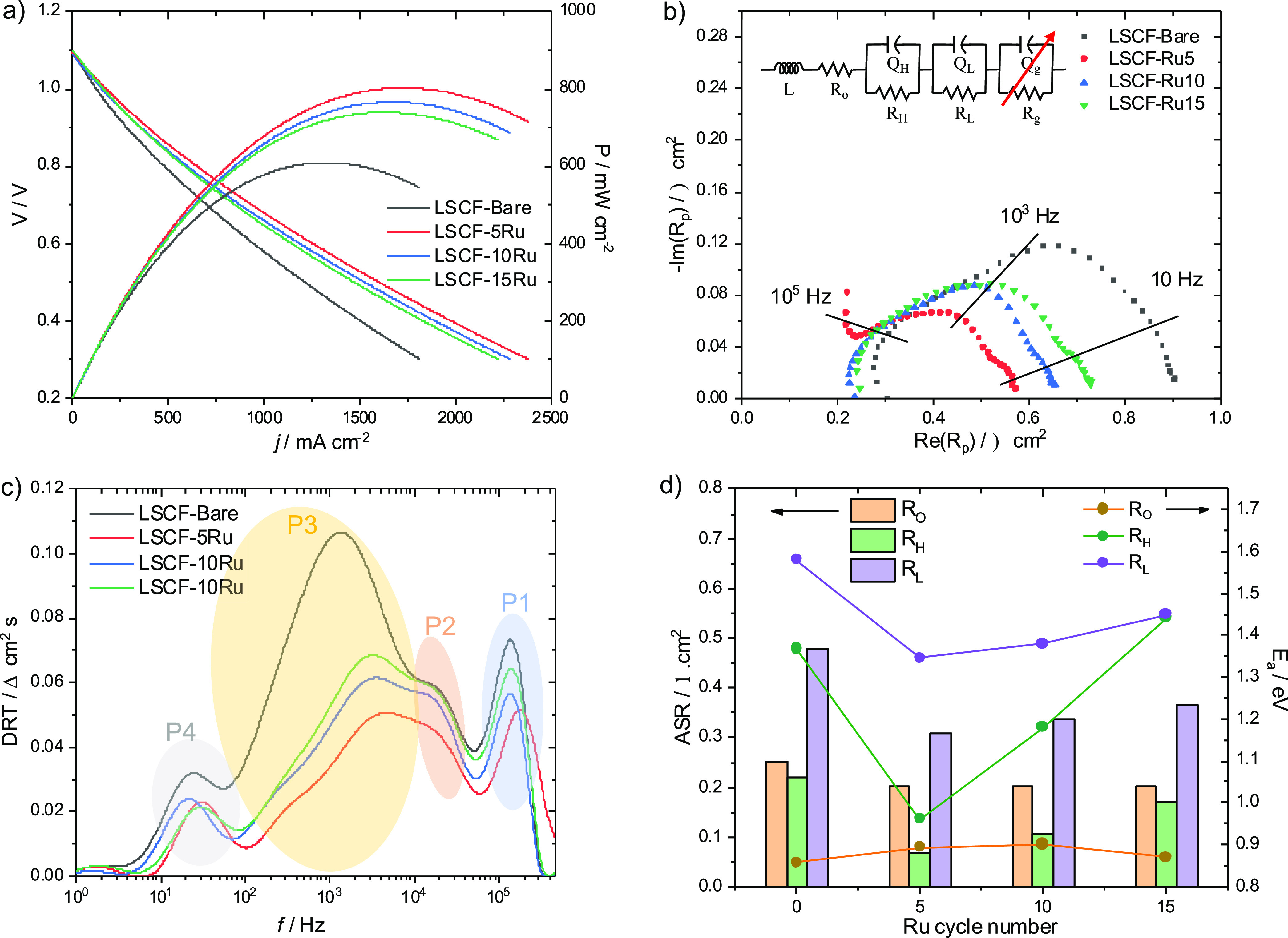
Electrochemical tests of as-prepared cells in fuel cell mode. (a) Polarization curves and power density curves. (b) Nyquist plots obtained at the cell voltage of 1.1 V and the equivalent circuit used for fitting. (c) DRT plots and (d) the fitted area specific resistances (ASR), including Ohmic resistances (*R*_O_) and polarization resistances (*R*_H_, *R*_L_), and their activation energies (*E*_a_). All of the tests were performed at 700 °C.

We examined the Ru-overcoating effect on ORR performance as well. Figure 9 illustrates the electrochemical data obtained from both bare and Ru-overcoated samples in fuel cell mode at 700 °C; the electrochemical data at different temperatures (660, 620, and 580 °C) are presented in Figure S7. The OCV closely aligns with the theoretical value of ∼1.1 V, providing evidence of gas tightness and minimal electronic crossover through the electrolyte. Notably, the additional Ru overcoat significantly improves the initial cell performance, as indicated in Figure 9a. Compared to the cell with bare LSCF as the electrode, cells with 5, 10, and 15 cycles of Ru overcoat by PEALD show enhancements in power density, with maximum values of 803, 766, and 739 mW cm^–2^, respectively, compared to 609 mW cm^–2^ for LSCF-Bare. LSCF-5Ru exhibits a 31.9% increase in performance, highlighting the effectiveness of an angstrom-level Ru overcoat. However, thicker Ru overcoats lead to gradual performance reduction.

EIS analysis was performed using the equivalent circuit shown in Figure 9b. Similar to the electrolysis mode, *R*_O_, *R*_H_//Q_H_, and *R*_L_//*Q*_L_ represent Ohmic transport, charge transfer, and oxygen adsorption processes, respectively. R_g_//Q_g_ in the *f*_c_ range of 10^1^–10^2^ Hz was added to model the gas transport (or mass transport) process within porous electrodes. Since the activation energy (*E*_a_) for *R*_O_ is very similar for all samples (0.87–0.9 eV), regardless of the Ru overcoat, the decrease in *R*_O_ can be attributed to enhanced connectivity between the electrolyte and the electrode. Regarding the electrode kinetics represented by *R*_H_ and *R*_L_, LSCF-5Ru shows the most enhanced performance. By performing 5 cycles of Ru ALD (corresponding to a 7.5 Å Ru overcoat), the electrode resistances were significantly reduced (*R*_H_: 0.22 → 0.07 Ω cm^2^; *R*_L_: 0.48 → 0.31 Ω cm^2^ at 700 °C), and their corresponding *E*_a_ values also were decreased (*E*_a_ for *R*_H_: 1.37 → 0.96 eV; *E*_a_ for *R*_L_: 1.58 → 1.34 eV), as shown in Figure 9d. The Arrhenius plots for the four samples are presented in Figure S8, and the fitted parameters are listed in Table S1. Similar to electrolysis mode, both the P2 and P3 processes in the DRT plot (Figure 9c), corresponding to *R*_H_//Q_H_ and *R*_L_//*Q*_L_ in the equivalent circuit, respectively, are highly facilitated by the Ru overcoats, further supporting the significant impact of surface-specific Ru on the electrode performance in the kinetics of ORR charge transfer and oxygen adsorption.

## Conclusions

In this report, we presented how an ultrathin layer of metallic Ru (7.5–22.5 Å) deposited onto porous LSCF via PEALD affects the performance of an air electrode (in both fuel cell and electrolysis modes) and durability (in electrolysis mode). It is demonstrated that an angstrom-level Ru overcoat significantly enhances electrode performance for both oxygen reduction and evolution reactions. The improved electrode activity by Ru ALD can be attributed to the substantial facilitation of both charge transfer and oxygen adsorption/desorption kinetics. We additionally presented that a Ru overcoat brings a dramatic benefit of maintaining oxygen desorption kinetics, along with the advantage of suppressing Sr segregation, during electrolysis mode operation. It was revealed that the deposited Ru reacts with surface Sr species to form SrRuO_3_, which is likely the main contributor to suppressing Sr segregation while maintaining a decent OER performance.

## Supporting Information Available

Additional experimental data of electrochemical and physicochemical properties (PDF)

## Abbreviations

DRT: distribution of relaxation time
EIS: electrochemical impedance spectroscopy
OER: oxygen evolution reaction
ORR: oxygen reduction reaction
SOEC: solid oxide electrolyzer cell
SOFC: solid oxide fuel cell
PEALD: plasma-enhanced atomic layer deposition
