# Supplementary material for: Ultralow Loading of Ru as a Bifunctional Catalyst for the Oxygen Electrode of Solid Oxide Cells
Source: ACS Catal. 2023 Aug 8;13(16):11172–81. doi: 10.1021/acscatal.3c02544 (PMC10442917; doi:10.1021/acscatal.3c02544)
Supplement: Supplementary file 1 — cs3c02544_si_001.pdf [file cs3c02544_si_001.pdf]

Supporting Information

Ultra-low Loading of Ru as a Bifunctional Catalyst for the Oxygen Electrode of Solid Oxide Cells

Haoyu Li[a,†], *Hyong June Kim*[b,†], *ThomasJae Garcia*[a], *Geonwoo Park*[b], *Yong Ding*[c], *Meilin Liu*[c], *Jihwan An*[d,*], *Min Hwan Lee*[a,*]

[a]Department of Mechanical Engineering, University of California, Merced, California 95343, United States

[b]Department of Manufacturing System and Design Engineering, Seoul National University of Science and Technology, Seoul 01811, Republic of Korea

[c]School of Materials Science and Engineering, Georgia Institute of Technology, Atlanta, Georgia 30332-0245, United States

[c]Department of Mechanical Engineering, Pohang University of Science and Technology (POSTECH), Pohang 37673, Republic of Korea

*E-mail for M.L.: mlee49@ucmerced.edu. * E-mail for J.A.: jihwanan@postech.ac.kr

Estimation of Ru wt.% of LSCF-5Ru

Given the complex geometrical properties of the $(La_{0.6}Sr_{0.4})_{0.95}Co_{0.2}Fe_{0.8}O_{3-\delta}$ (LSCF) electrode, we employed a simple method to estimate the weight percentage of Ru in the LSCF-5Ru. This estimation is based on two relationships, as follows:

$$V\%(Ru) = \frac{\pi D^2 \cdot t}{\pi D^3/6} \tag{1}$$

$$wt\%(Ru) = \frac{6 \cdot t}{D} * \frac{\rho_{Ru}}{\rho_{LSCF}} \tag{2}$$

where *t*, *D*, V%, and wt.% correspond to the nominal thickness of Ru overcoat, the median diameter of LSCF particles on the air electrode, volume percentage, and weight percentage, respectively. The median diameter of LSCF particles has been determined as 680 nm, according to the characteristic data from the vendor (FuelCellMaterials). It is noted that in Equation (1), we presumed that (a) the LSCF particles to be perfect spheres, (b) there is no loss of surface area from the overlaps of neighboring LSCF particles; (c) the ALD-based Ru is uniformly coated throughout the all the surface of LSCF particles. This suggests that the actual weight percentage of Ru in LSCF-5Ru should be substantially lower than the calculated value.

The density of Ru is 12.2 g cm^{-3}, while that of LSCF is 6.08 g cm^{-3} (with 40% Sr doped on the A-site).[1] Given these densities and the estimated nominal thickness of Ru overcoat on LSCF-Ru (7.5 Å), we conclude that the highest possible estimated weight percentage for Ru is 1.33%.

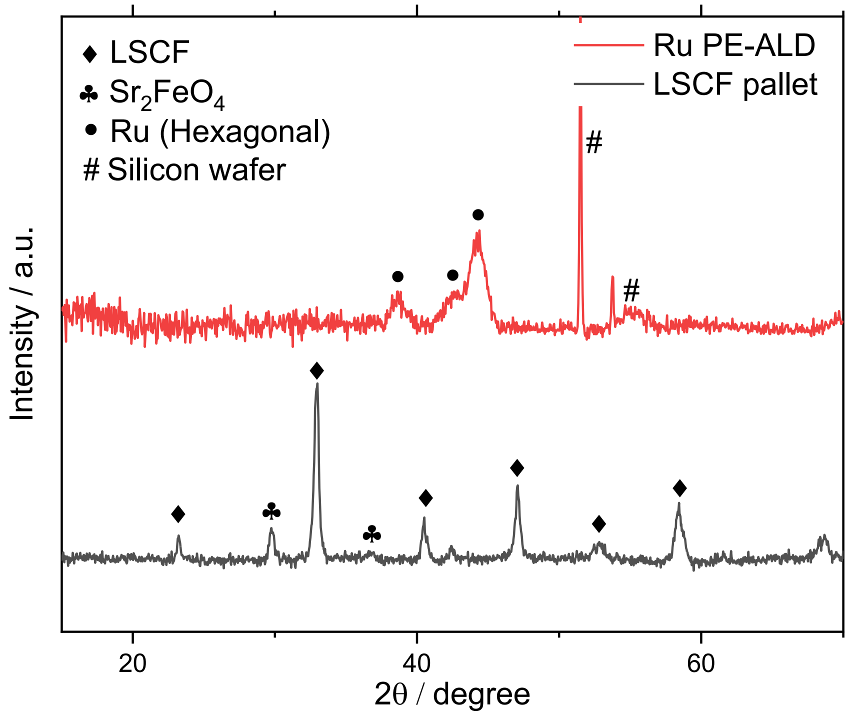

Figure S1. XRD spectra of Ru and LSCF. The Ru sample is prepared by performing 300 cycles of Ru PE-ALD on a Si wafer. The LSCF sample is prepared by pressing LSCF powders, followed by a sintering process as detailed in the Experimental section.

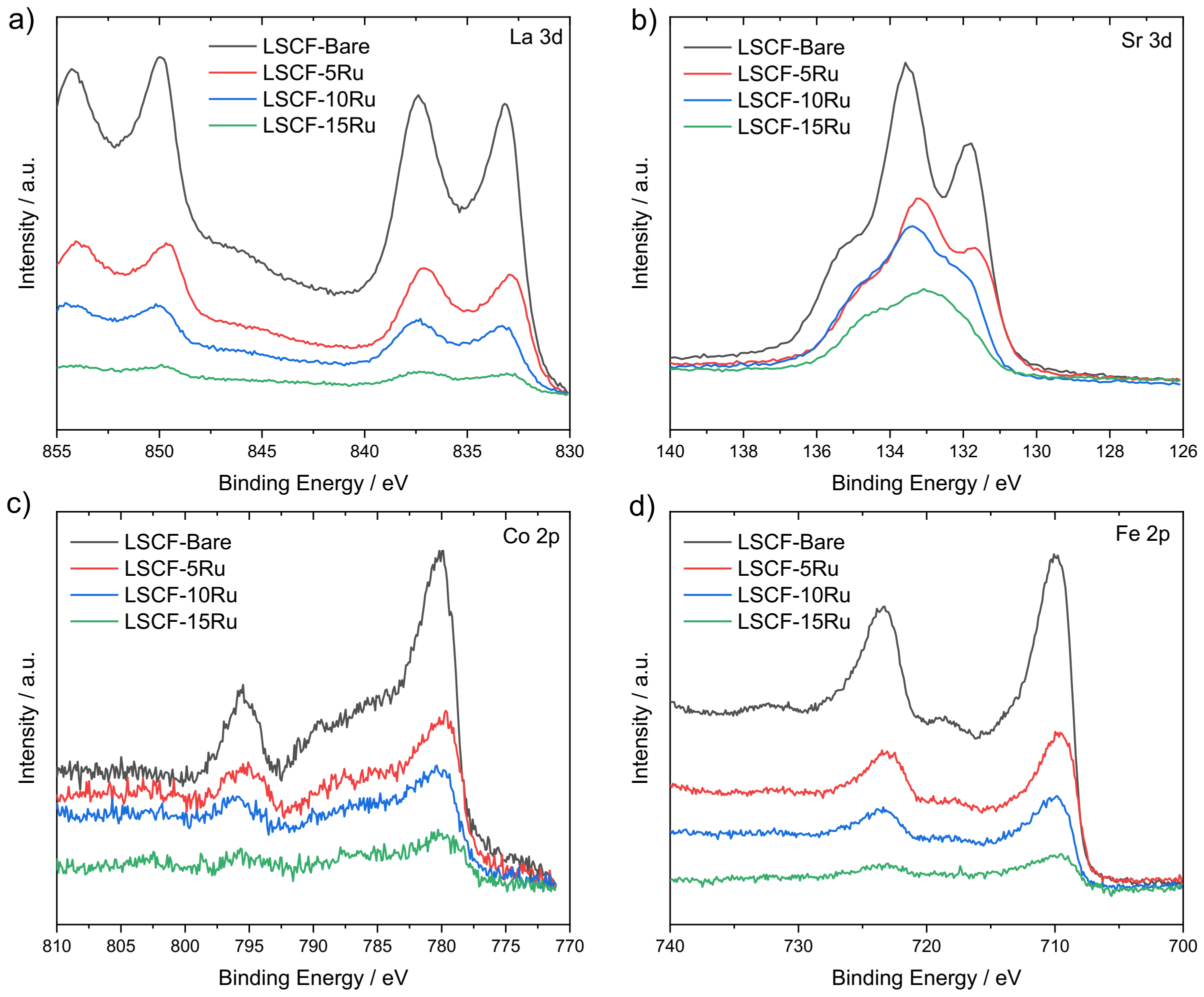

Figure S2. XPS spectra of (a) La 3d, (b) Sr 3d, (c) Co 2p, and (d) Fe 2p obtained from the as-prepared cells. It is observed that the intensities of all the cations decrease with Ru overcoat thickness.

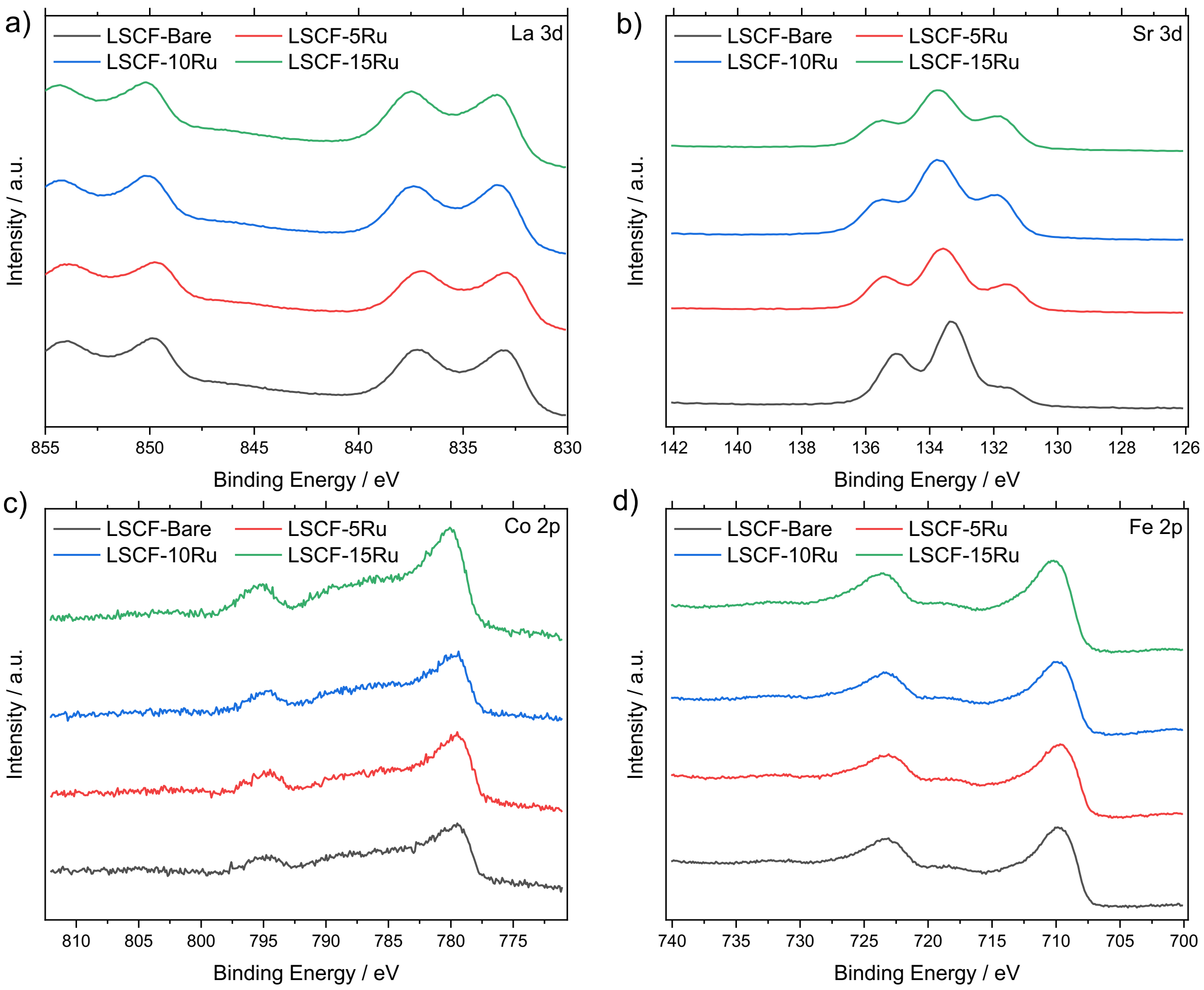

Figure S3. XPS spectra of (a) La 3d, (b) Sr 3d, (c) Co 2p, and (d) Fe 2p obtained from the post-operation cells that underwent the 90-h long operation in electrolysis mode.

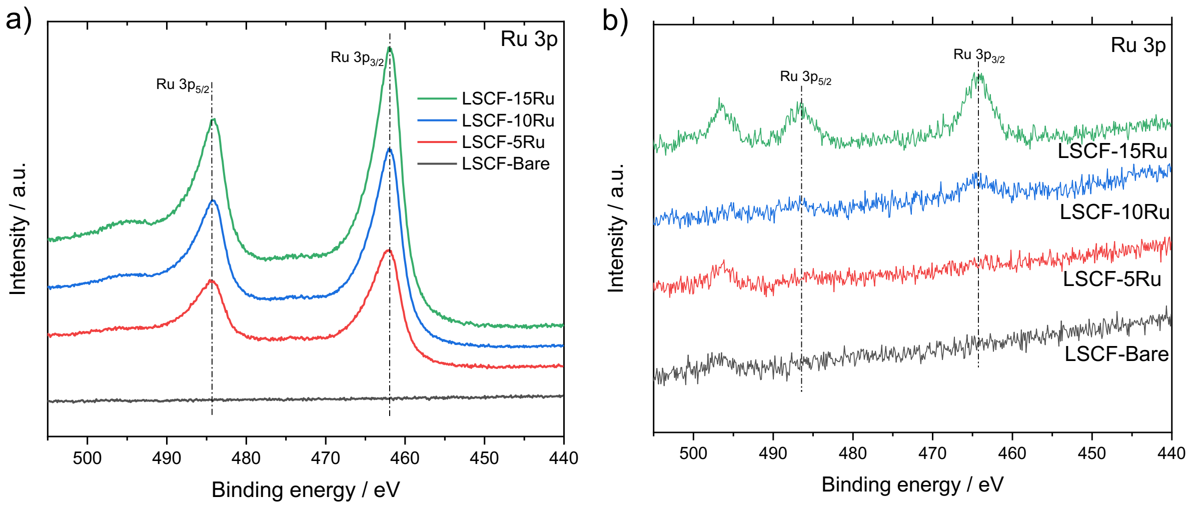

Figure S4. Ru 3p XPS spectra (a) before and (b) after the 90-h operation in the electrolysis mode. The presence of Ru species in the as-prepared and post-operation cells was determined using Ru 3p spectra, as the Ru 3d spectra overlap with C 1s spectra, thus making them difficult to discern. A notable decrease in Ru species is observed after the 90 h-long operations as shown in (b). There are two potential explanations for this observation. Firstly, the decrease could be due to the strong oxidation of Ru and subsequent evaporation of the oxidized Ru during operation; the evaporation point of ruthenium oxide is significantly lower than 700 °C, the operating temperature.[2] Secondly, metal species on the electrode surface may have migrated into the bulk of the material.[3]

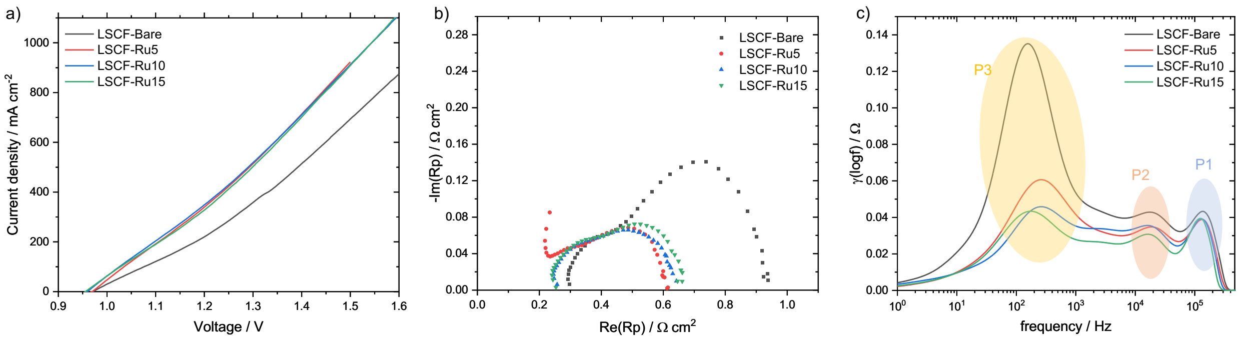

Figure S5. Electrochemical tests of post-operation cells in the electrolysis mode. (a) Polarization curves, (c) EIS curves obtained at 0.97 V along with the equivalent circuit used for fitting, and (d) DRT curves. All the tests were performed at 700 ºC. The cells have undergone the 90-h durability test in electrolysis before the measurements presented here.

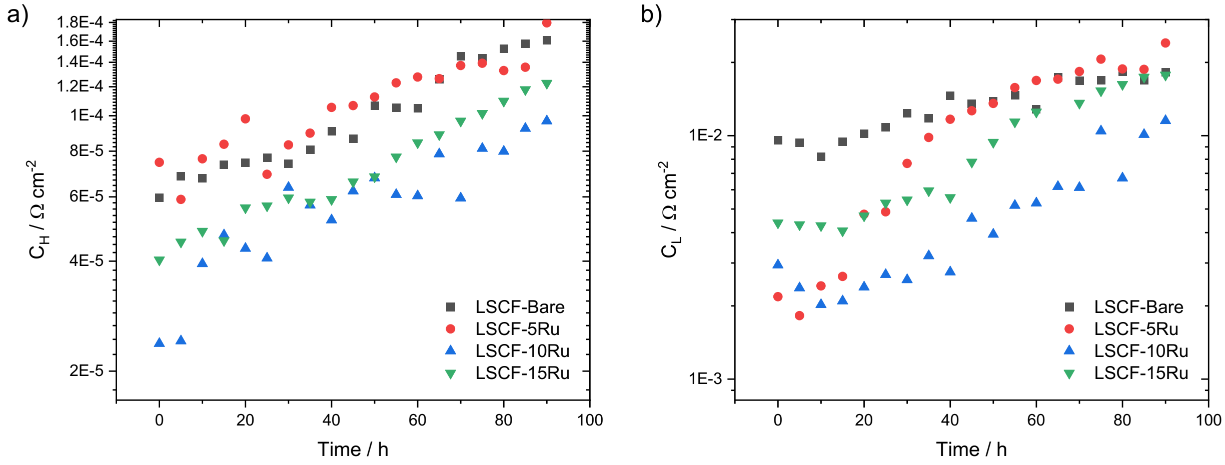

Figure S6. Pseudo-capacitance values, C_H and C_L, quantified from EIS measurements at different stages of cell durability test in electrolysis mode at 500 mA cm^{-2}. The EIS measurements were performed at 0.97 V.

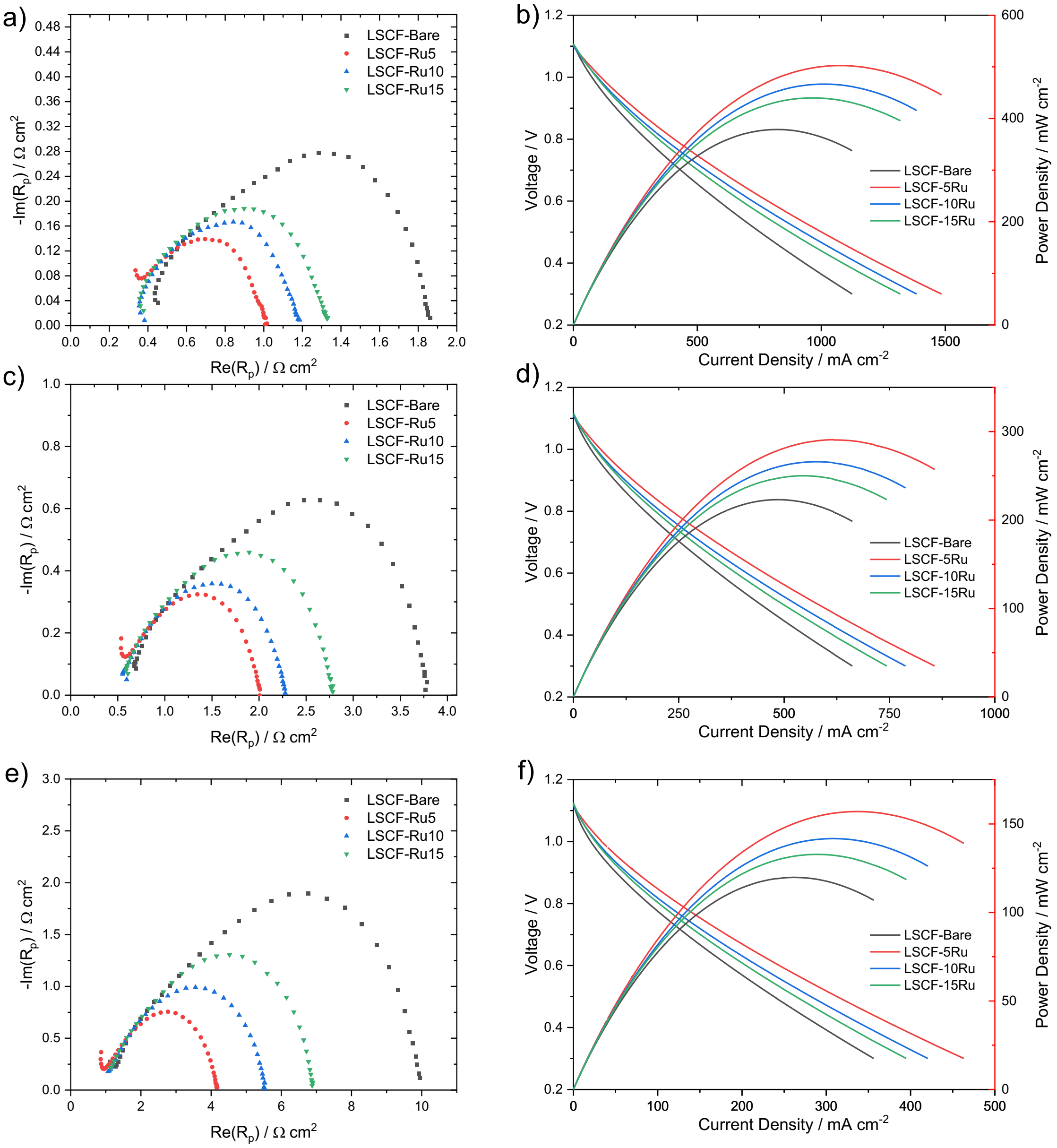

Figure S7. Nyquist plots and polarization curves (along with power density curves) obtained at (a,b) 660 ºC, (c,d) 620 ºC, and (e,f) 580 ºC.

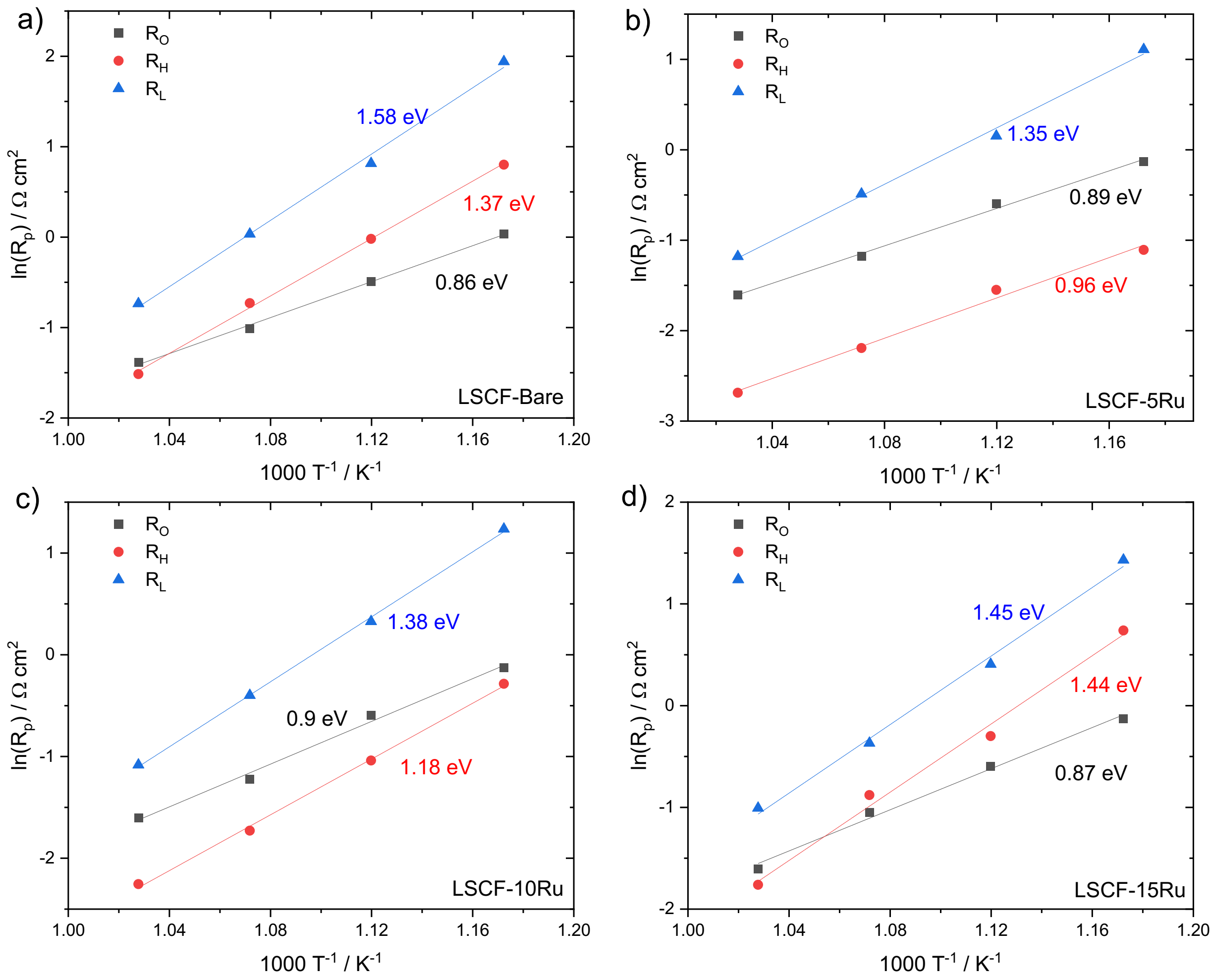

Figure S8. Arrhenius plots of R_O, R_H, and R_L with their corresponding activation energies for (a) LSCF-Bare, (b) LSCF-5Ru, (c) LSCF-10Ru, and (d) LSCF-15Ru. The values are deduced from EIS measurements at 1.1 V in fuel cell configuration; the values are tabulated in Table S1.

Table S1. Resistance and pseudo-capacitance values deduced from EIS data, which is performed in fuel cell mode at 1.1 V.

Temperature [°C]	Sample	R_{ohm} [Ω cm^2]	R_H [Ω cm^2]	C_H [μF cm^{-2}]	R_L [Ω cm^2]	C_L [mF cm^{-2}]
700	LSCF-Bare	0.25	0.22	27.52	0.48	1.56
	LSCF-5Ru	0.20	0.07	45.27	0.31	0.67
	LSCF-10Ru	0.20	0.11	40.74	0.34	1.07
	LSCF-15Ru	0.20	0.17	24.00	0.37	0.82
660	LSCF-Bare	0.36	0.48	21.94	1.03	1.09
	LSCF-5Ru	0.31	0.11	25.15	0.61	0.23
	LSCF-10Ru	0.30	0.18	23.43	0.67	0.35
	LSCF-15Ru	0.30	0.42	18.34	0.69	0.46
620	LSCF-Bare	0.61	0.98	19.43	2.26	0.67
	LSCF-5Ru	0.55	0.21	43.26	1.16	0.22
	LSCF-10Ru	0.55	0.35	28.55	1.39	0.30
	LSCF-15Ru	0.55	0.74	21.88	1.50	0.41
580	LSCF-Bare	1.04	2.23	15.05	6.97	0.39
	LSCF-5Ru	0.88	0.33	17.08	3.03	0.13
	LSCF-10Ru	0.88	0.75	33.18	3.44	0.24
	LSCF-15Ru	0.88	2.09	42.02	4.19	0.31

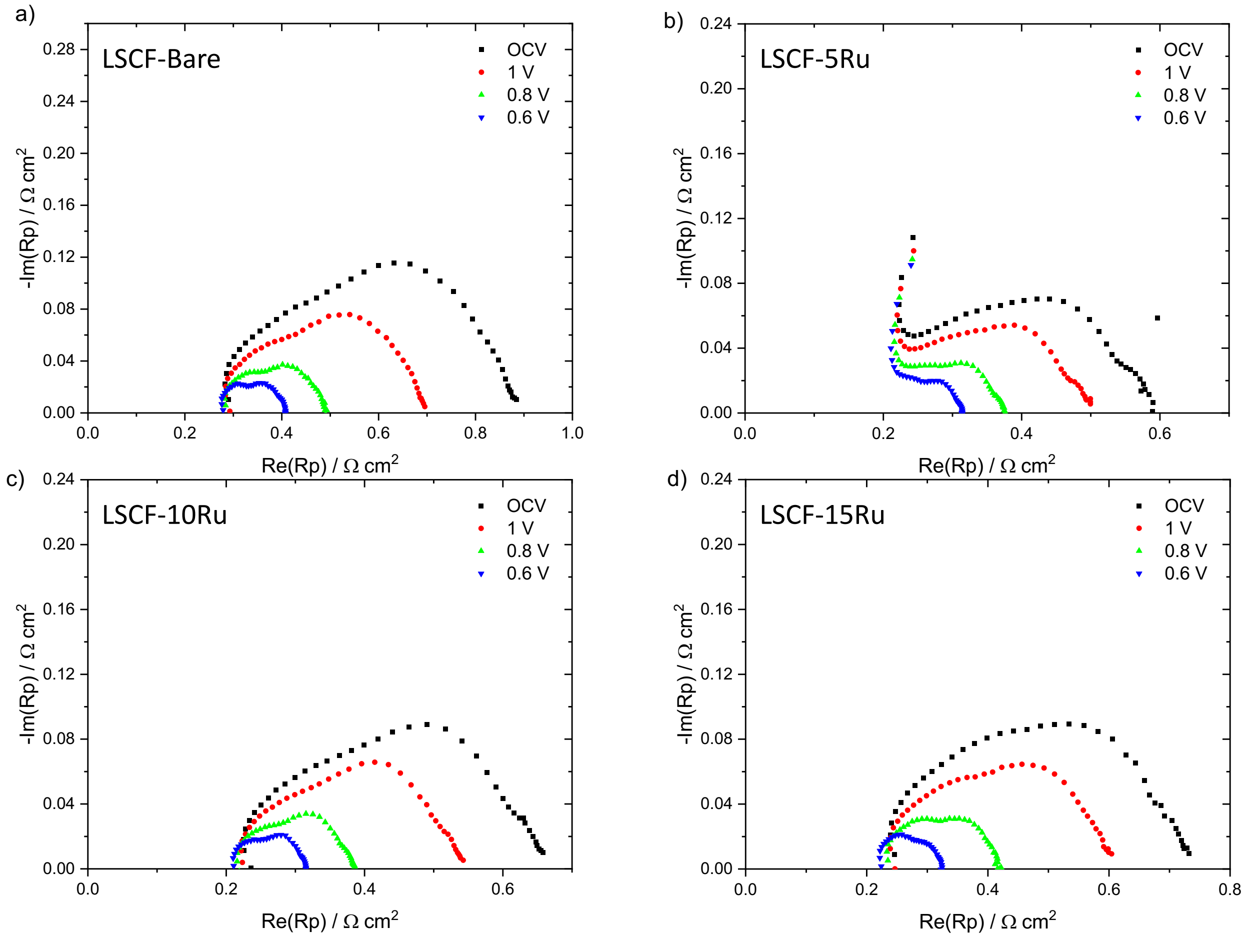

Figure S9. Nyquist plots obtained from (a) LSCF-Bare, (b) LSCF-5Ru, (c) LSCF-10Ru, and (d) LSCF-15Ru in fuel cell mode at 700 ºC, at different potentials of OCV, 1.0 V, 0.8 V and 0.6 V.

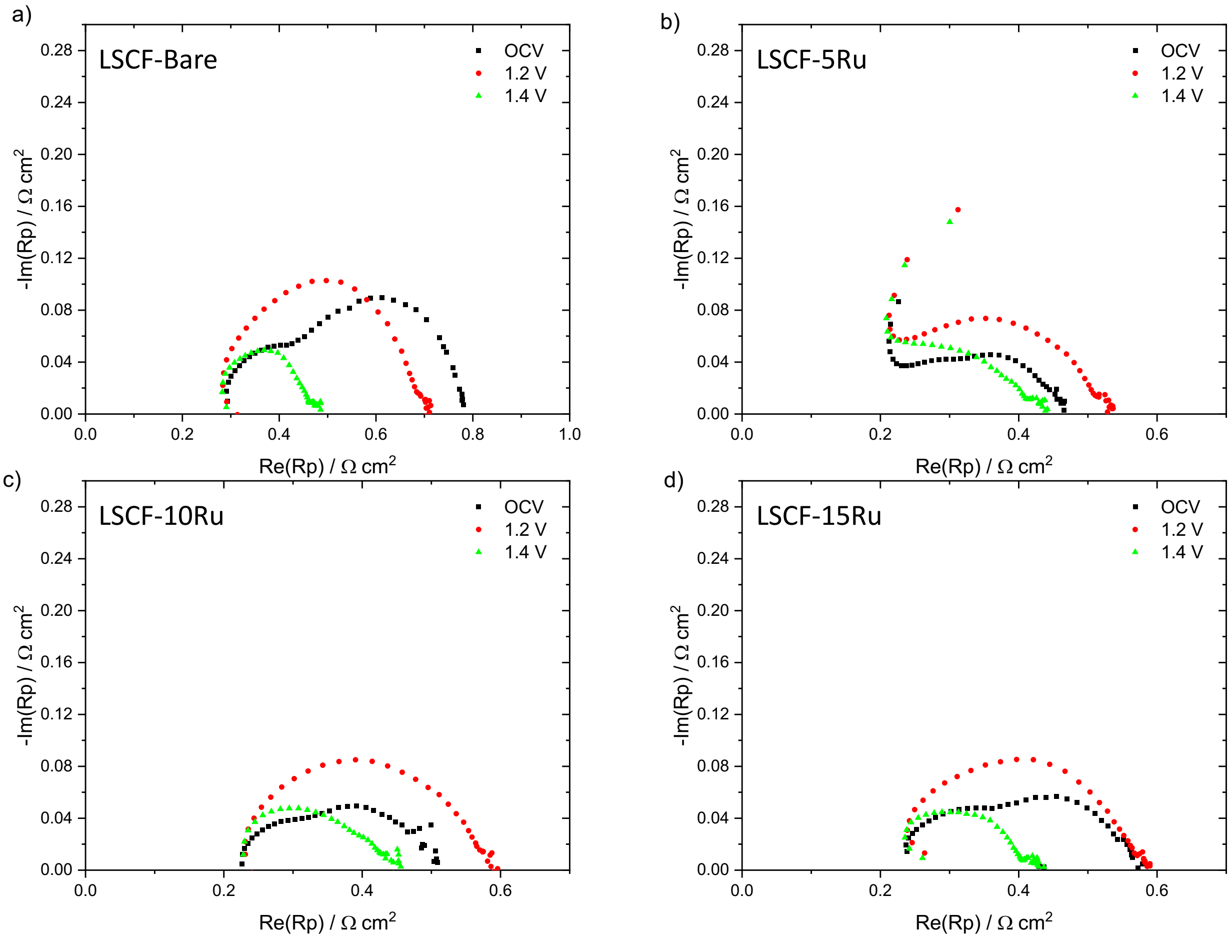

Figure S10. Nyquist plots obtained from (a) LSCF-Bare, (b) LSCF-5Ru, (c) LSCF-10Ru, and (d) LSCF-15Ru in electrolysis mode at 700 ºC, at different potentials of 0.97 V, 1.2 V, and 1.4 V.

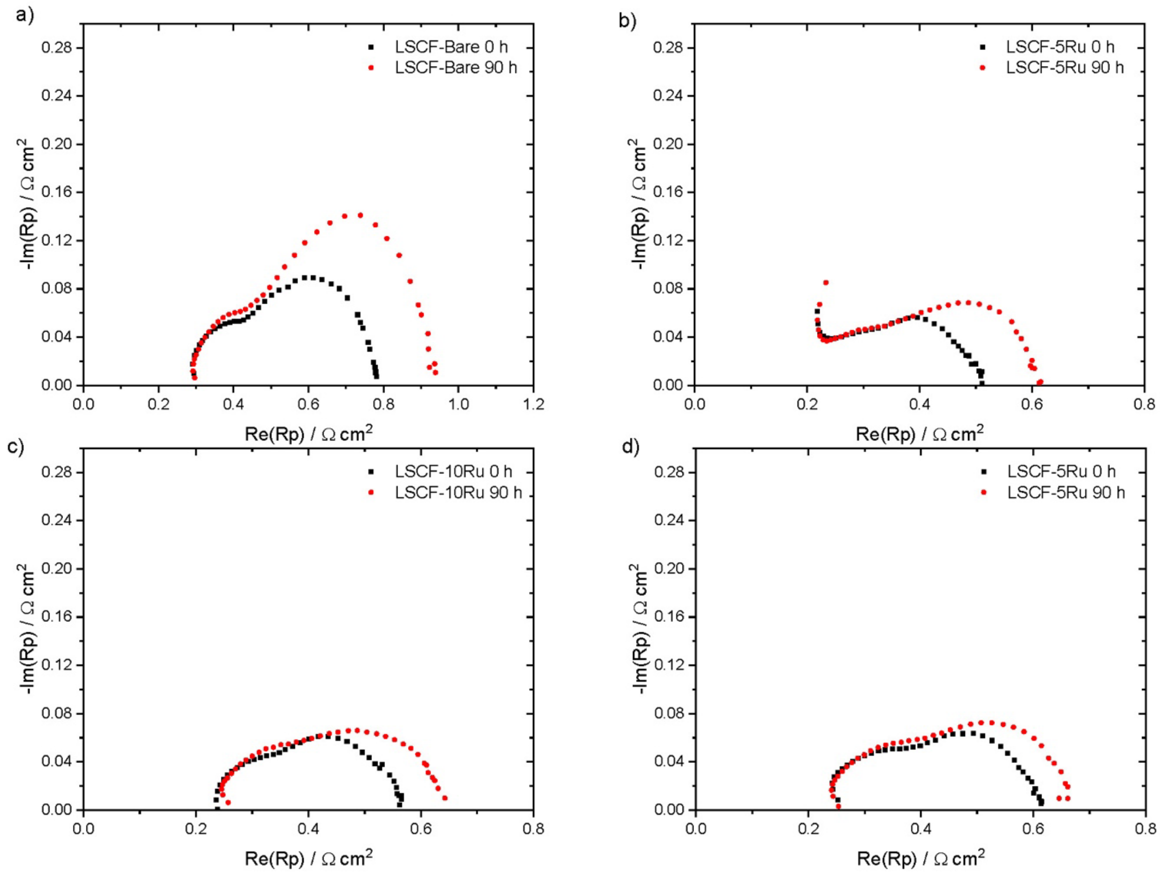

Figure S11. Nyquist plots obtained from (a) LSCF-Bare, (b) LSCF-5Ru, (c) LSCF-10Ru, and (d) LSCF-15Ru in electrolysis mode at 700 ºC at the initial stage (0 h) and the final stage (90 h) in the durability test.

Reference

(1) Chou, Y.-S.; Stevenson, J. W.; Armstrong, T. R.; Pederson, L. R. Mechanical Properties of La1-XSrxCo0.2Fe0.8O3 Mixed-Conducting Perovskites Made by the Combustion Synthesis Technique. *J. Am. Ceram. Soc.* **2000**, *83* (6), 1457–1464. https://doi.org/10.1111/j.1151-2916.2000.tb01410.x.

(2) Koda, Y. Boiling Points and Ideal Solutions of Ruthenium and Osmium Tetraoxides. *J. Chem. Soc. Chem. Commun.* **1986**, No. 17, 1347–1348. https://doi.org/10.1039/C39860001347.

(3) Shin, S. M.; Yoon, B. Y.; Kim, J. H.; Bae, J. M. Performance Improvement by Metal Deposition at the Cathode Active Site in Solid Oxide Fuel Cells. *Int. J. Hydrog. Energy* **2013**, *38* (21), 8954–8964. https://doi.org/10.1016/j.ijhydene.2013.04.115.
